# Network analysis of microRNAs and their regulation in human ovarian cancer

**DOI:** 10.1186/1752-0509-5-183

**Published:** 2011-11-03

**Authors:** Sebastian Schmeier, Ulf Schaefer, Magbubah Essack, Vladimir B Bajic

**Affiliations:** 1 Computational Bioscience Research Center (CBRC), 4700 King Abdullah University of Science and Technology (KAUST), Thuwal 23955-6900, Kingdom of Saudi Arabia

## Abstract

**Background:**

MicroRNAs (miRNAs) are small non-coding RNA molecules that repress the translation of messenger RNAs (mRNAs) or degrade mRNAs. These functions of miRNAs allow them to control key cellular processes such as development, differentiation and apoptosis, and they have also been implicated in several cancers such as leukaemia, lung, pancreatic and ovarian cancer (OC). Unfortunately, the specific machinery of miRNA regulation, involving transcription factors (TFs) and transcription co-factors (TcoFs), is not well understood. In the present study we focus on computationally deciphering the underlying network of miRNAs, their targets, and their control mechanisms that have an influence on OC development.

**Results:**

We analysed experimentally verified data from multiple sources that describe miRNA influence on diseases, miRNA targeting of mRNAs, and on protein-protein interactions, and combined this data with *ab initio *transcription factor binding site predictions within miRNA promoter regions. From these analyses, we derived a network that describes the influence of miRNAs and their regulation in human OC. We developed a methodology to analyse the network in order to find the nodes that have the largest potential of influencing the network's behaviour (network hubs). We further show the potentially most influential miRNAs, TFs and TcoFs, showing subnetworks illustrating the involved mechanisms as well as regulatory miRNA network motifs in OC. We find an enrichment of miRNA targeted OC genes in the highly relevant pathways cell cycle regulation and apoptosis.

**Conclusions:**

We combined several sources of interaction and association data to analyse and place miRNAs within regulatory pathways that influence human OC. These results represent the first comprehensive miRNA regulatory network analysis for human OC. This suggests that miRNAs and their regulation may play a major role in OC and that further directed research in this area is of utmost importance to enhance our understanding of the molecular mechanisms underlying human cancer development and OC in particular.

## Background

MicroRNAs (miRNAs) are short (~22 nucleotides) non-coding RNA molecules, which influence gene expression mainly through degradation of coding mRNA [1,2]. Similar to protein coding genes, miRNA genes (pri-miRNAs) thus far identified are predominantly transcribed by RNA polymerase II [3,4]. New evidence, however, also indicates the involvement of RNA polymerase III for certain miRNAs [5,6]. These findings suggest that pri-miRNAs are regulated on the transcriptional level in a similar fashion as protein coding genes. Following transcription, pri-miRNAs are cleaved through the microprocessor complex *Drosha *and *DGCR8 *to generate 60~70nt pre-miRNAs [7,8]. *Exportin-5 *and its co-factor *RanGTP *then export the pre-miRNAs into the cytoplasm [9], where they are cleaved by *Dicer*, an RNase III endonuclease, to yield the mature miRNAs [2,10].

A wide range of mRNAs are targeted by miRNAs. Numerous databases [11-15], such as the TarBase database [16] and miRTarBase [17], hold experimentally validated or computationally predicted miRNA targets. Consequently, recent experimental and computational studies focus on the regulatory regions or transcription start sites (TSSs) of miRNA genes [18-22], their associated transcription factors (TFs) [23-25], and their effects on miRNA transcription [23]. Nevertheless, the signals and mechanisms that govern miRNA transcriptional regulation remain unclear.

Interest in miRNAs and their control is governed by the knowledge that a multitude of miRNAs are implicated in a variety of human diseases [26-28], including several human cancers [29,30]. Recent studies and reviews focused on miRNA involvement in the progression of the most serious gynaecological cancer, ovarian cancer (OC), and it is now commonly accepted that miRNAs play a role in OC [31-34]. Experimental data shows that several miRNAs exhibit altered expression levels in OC cell lines [35-37]. Nevertheless, knowledge about possible miRNA regulatory roles within OC initiation and progression is incomplete. The transcriptional regulation of the pri-miRNAs involved in OC is an important process which, if understood, could positively impact current treatments and survival rates of patients with OC.

In the present study, we investigated the underlying network of miRNAs, their targets, and their control mechanisms that are associated with OC development. Changes in miRNA networks between cancerous and healthy tissue have recently been illuminated [38]. Here we concentrate on miRNAs and their regulation in OC. We considered all human miRNAs that are listed in miRBase [15]. For this set of miRNAs, we extracted experimentally verified targets from TarBase [16] and miRTarBase [17] and selected those that have been associated with OC in accordance with the Dragon Database for Exploration of Ovarian Cancer Genes (DDOC, [39]). In addition, we mapped transcription factor binding sites (TFBSs) onto extracted promoter regions of the human miRNAs from miRBase to establish a link between TFs and miRNAs. We considered two different sizes of promoter regions, 1000nt and 5000nt upstream of the miRNA gene body. In addition, we incorporated human curated protein-protein interaction (PPI) data from several interaction databases [40-44] to identify other proteins that may partake indirectly in the regulation of the miRNAs and to establish links between all participating proteins in the network.

From these considerations, we derived two large networks (one for each considered promoter size) of interacting biological entities (nodes) that are centred on the miRNAs and that impact OC. We combined miRNAs, their downstream targets, and their upstream regulatory proteins in these networks in order to place these entities in the context of OC. Subsequently, we devised a methodology to assign a rank to each node according to its potential for influencing the network's behaviour. This rank is based on the number of potential interaction partners that a network node might have as well as the type of interaction a node engages in. Finally we identified regulatory network motifs in the networks. We defined regulatory network motifs to be the smallest possible group of nodes in the network that form a closed circle of interactions with each other. We propose that these network motifs are integral constituent parts of the network and the main stepping-stones that can be used to further an understanding of the networks behaviour, as well as potential targets for possible attempts to interfere with the network's behaviour.

The results of this network analysis show that miRNAs and their regulation play an important role within OC and further in-depth research in this direction may be rewarding.

## Results and discussion

### MicroRNAs and their targets involved in OC

The focus of our study is to contribute to the current knowledge of human miRNA involvement in OC. As a starting point we used all human miRNAs that are listed in miRBase [15]. For these we extracted all experimentally confirmed miRNA targets from TarBase [16] and miRTarBase [17] and subsequently restricted our analysis to those miRNAs that have an experimentally confirmed target that is involved in the progression of OC according to the DDOC database [39]. In total we extracted 162 miRNAs targeting 131 different human proteins. DDOC lists a total of 379 genes that are relevant for the progression of OC. This means that more than one third of the OC genes indentified so far are experimentally confirmed targets of miRNAs. TarBase and miRTarBase together list no more than 1800 human genes as confirmed targets of miRNAs. This means that miRNA targets are overrepresented among OC genes with regard to all human genes. While an explanation for this observation remains elusive, it merits a closer examination of the role of miRNAs and their regulation in OC.

Research by Laios *et al*. [45] suggests that various miRNAs are involved in different stages of cancer progression. Among the OC-relevant proteins that are experimentally proven to be targeted by these miRNAs are several proteins that are key members of important cancer pathways. Two examples are that *miR-214 *induces cell survival and cisplatin resistance through targeting the 3'-untranslated region (UTR) of PTEN, which leads to down-regulation of PTEN and the activation of the Akt pathway [37]. PTEN is also targeted by 13 other miRNAs. Furthermore, *miR-15b *and *miR-16 *inhibit BCL2 expression thereby initiating cleavage of pro-caspase 9 and PARP and consequently leading to apoptosis [46]. BCL2 is targeted by a total of 23 miRNAs.

### MicroRNAs and their regulatory proteins

To increase our understanding of how the 162 miRNAs identified above are regulated on the transcriptional level, we extracted the promoter region sequences of the miRNA genes that correspond to the 162 miRNAs identified above from the UCSC database [47]. Two different promoter lengths of 1000nt and 5000nt upstream of the reported miRNA gene were extracted thereby ensuring that one component of our analysis focuses on the transcriptional elements in the core promoter while the other considers a more comprehensive set of *cis*-regulatory elements operating at greater distance. Differences in the resulting networks will aid in highlighting the interactions of regulatory proteins with regulatory motifs that are primarily located in the core promoter as well as regulatory processes that include more distally located regulatory sequence motifs. To do this, we mapped BIOBASE TRANSFAC binding site motifs to the promoter regions [48] and linked TFs to the mapped TFBSs (see Methods). We additionally extracted high-confidence transcription co-factors (TcoFs) that interact with these TFs from TcoF-DB [49]. We found that 237 TFs and 140 high-confidence TcoFs may be involved in the core regulation (1000nt upstream) of the 162 OC-associated miRNAs. When examining the larger and potentially more comprehensive set of TFBS (5000nt upstream of the miRNA genes), 244 TFs were predicted to bind to the extended promoter regions of OC-associated miRNAs. The seven additionally considered TFs (GATA6, LHX3, MTF1, NFIL3, NKX31, ZBTB6, ZN350) only have predicted binding sites that are more distally located from the TSS and do not interact with any new high-confidence TcoFs.

### Network construction

To generate a network, we extracted from PPI data (see Methods) all interactions for the targets of all 162 OC-associated miRNAs, relevant TFs, and TcoFs. After we combined all the interactions we derived two networks of interest, one for core promoter regulatory elements (NW1000) and one for the more comprehensive set of regulatory elements (NW5000). Table 1 shows the numbers of various types of nodes and numbers of interactions (edges) in the two networks. The complete networks can be found in the Additional Files 1 and 2. Including PPIs, transcriptional regulation and miRNA targeting the role of miRNAs in OC is seen as a complex and highly interconnected network.

**Table 1:** Number of nodes in the networks of OC-associated elements NW1000 and NW5000

	NW1000	NW5000
**miRNAs**	162	162

**miRNA targets (OC cancer genes)**	131	131

**Transcription factors**	237	244

**Transcription co-factors**	140	140

**Edges miRNA→ target**	434	434

**Edges TF → miRNA**	5327	14720

**Protein-protein-interactions**	4382	4409

**Total edges**	10143	19563

**Total nodes**	651	658

The table shows the number of different nodes in the constructed networks. NW1000 concentrates on regulatory elements in the core promoter while NW5000 attempts to also include more distally located regulatory elements.

### Network hubs

To identify those components of the networks that have the most potential to influence to networks' overall behaviour, we implemented an edge-based ranking system. Each node is ranked according to a score based on its outgoing edges of the first and second degree (see Methods). However, there are three different types of edges within the network, two directed edge types, namely miRNA to target association, and TF to miRNA association, and an undirected edge of the type PPI. The latter is assumed to have the least influence on the information flow within the network. We assume that the most important edge in the network *e*_t _is the interface between a miRNA and its target, because this is a directed edge that has been experimentally proven. The second most important edge *e*_r _is an association between a TF and a miRNA, even though only predicted, has a direction. The least important edge *e*_i _is the undirected interaction between two proteins.

This means that each node in the network is ranked based on the number of potential binding partners as well as on the type of molecular interaction that it engages in. Our networks thus constitute a model that attempts to describe mechanisms in the living cell in the form of a weighted directed graph. Other aspects of a living cell, such as expression levels of the genes involved or the current developmental stage of the cell, are not part of our model.

Applying our weighting algorithm (see Methods) to the two networks NW1000 and NW5000 generated a ranking for each node. The complete node ranking for all nodes in NW1000 and NW5000 can be found in the Additional Files 3 and 4. Here we highlight nodes that were ranked high in both networks. We interpret these nodes as network hubs, potentially having the greatest influence on the regulation of miRNAs involved in OC.

It is known that elements located far upstream from a gene can contribute to its regulation [50,51]. However, when sizing a gene's upstream region to study its promoter region one has to more or less arbitrarily set a length limit. Any sequence length chosen is always a trade-off between excluding elements further upstream that might be relevant (shorter length) and including them alongside irrelevant DNA in the analysis introducing noise (longer length). While aware of these unavoidable shortcomings, we included two lengths that are frequently chosen as a compromise.

TFs that are ranked more highly in NW1000 can be understood to have a higher concentration of binding sites close to the start of the miRNA genes, while those that are ranked higher in NW5000 can be interpreted as having a tendency to bind more distally from the gene body. Naturally, the TFs appearing in NW1000 are a subset of those appearing in NW5000, but the TFs that are exclusively in NW5000 can be understood to bind to more remote binding sites. Seven such TFs were identified (see above).

There is an overlap of six TFs between the nodes ranked 1^st ^to 10^th ^in both networks (BRCA1, SP1, ESR1, SMAD3, PO2F1, TFE2) and therefore we regard these six TFs as the essential regulatory elements for miRNA regulation in human OC. Together they are predicted to regulate 148 out of 162 miRNAs in NW1000, which in turn target 130 out of 131 experimentally validated miRNA target proteins. In NW5000, these six TFs participate in the regulation of all 162 OC relevant miRNAs. This means that when an upstream regulatory region of 5000nt is considered, these six TFs are predicted to participate in the regulation of all OC relevant miRNA genes and with that have a potential influence on the expression levels of all OC relevant genes whose mRNAs are targeted by a miRNA. To the best of our knowledge, BRCA1, SP1, TFE2, PO2F1 and ESR1 have not been experimentally validated as TFs of the identified OC relevant miRNAs. It has been demonstrated that ESR1 mediated a decrease in *hsa-mir-21 *expression correlated with increased protein expression of endogenous *hsa-mir-21 *targets such as PDCD4, PTEN, and BCL2 [52]. It has however not been validated that ESR1 mediated this process as a TF of *hsa-mir-21 *and no binding site for ESR1 is predicted in the *hsa-mir-21 *promoter region using our method. SMAD3 has been shown to bind and transcribe the *hsa-mir-24 *promoter during myoblast differentiation [53]. Also, SMAD3 has been implicated in other diseases by acting as a TF for other miRNAs, for example: SMAD3 drives *hsa-mir-192 *expression thereby mediating renal fibrosis [54] and SMAD3 has been shown to bind the *let-7d *promoter thereby promoting idiopathic pulmonary fibrosis [55].

**Figure 1:**
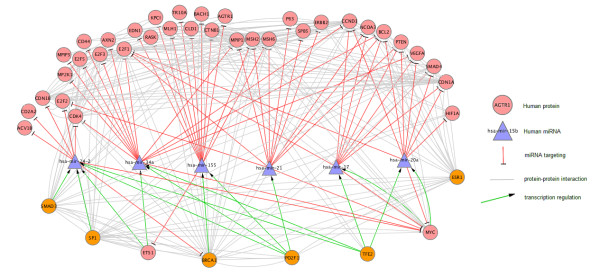
**Core network**. Large, highly connected subnetwork of NW1000 illuminating the interactions of six highly relevant TFs with six highly ranked miRNAs and their 38 OC relevant targets. For simplicity the TcoFs that interact with the TFs shown are omitted. The TFs BRCA1, SP1, ESR1, SMAD3, PO2F1 and TFE2 are ranked very high by our algorithm in both networks NW1000 and NW5000 and can be considered network hubs for miRNA regulation in human OC.

The highest ranking miRNAs according to our algorithm are *hsa-mir-20a, hsa-mir-24-2, hsa-mir-34a, hsa-mir-21, hsa-mir-17 *and *hsa-mir-155*. This is the first time that *hsa-mir-155 *has been linked to the progression of OC. However, recent research is focused on designing *hsa-mir-155 *based therapies as it has been demonstrated that a moderate increase in *hsa-mir-155 *levels is observed in many types of malignancies, and transgenic over-expression of the miRNA in mice results in cancer, whilst high levels of *hsa-mir-155 *expressed during immune response and hematopoietic lineage differentiation does not harm the organism [56]. It has recently been shown that *hsa-miR-20a *is differentially expressed in female oocytes [57]. In our networks *hsa-mir-20a *targets a total of nine genes that are implicated in the progression of OC. *miR-24 *has been found to regulate apoptosis in cancer cells and has already been suggested as a drug target for cancer therapy [58]. For graphical illustration, Figure 1 shows a large, highly connected subnetwork of NW1000 illuminating the interactions of six highly relevant TFs with six highly ranked miRNAs and their 38 OC relevant targets. For simplicity the TcoFs that interact with the TFs shown are omitted.

Among the TcoFs in the networks EP300, RB, UBC9, HDAC1, MED1, CTNB1, HDAC2, PML and NCOA6 are ranked high and therefore have a high potential of being more influential with regard to the networks' overall behaviour than others. These nine TcoFs appear in the list of best-ranked TcoFs of both networks NW1000 and NW5000. The human protein EP300 is the highest ranked TcoF in both networks, NW1000 and NW5000. It interacts with 51 TFs who in turn regulate all but five (all miRNAs in NW5000) OC-relevant miRNAs in NW1000.

**Figure 2:**
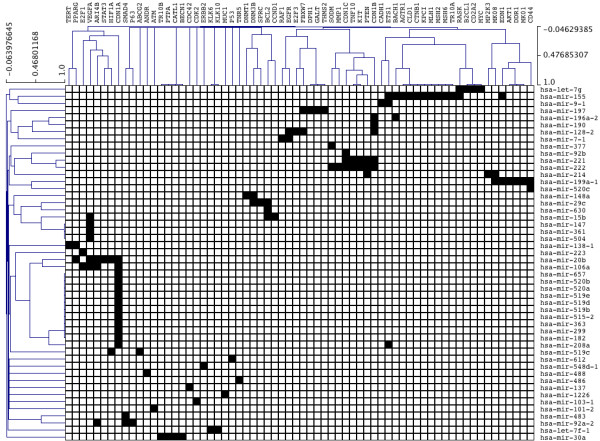
**Heatmap of a sub-network for significant TF BRCA1**. BRCA1 can be regarded as the most significant node for OC relevant miRNA regulation. It is ranked 1^st ^in both networks NW1000 and NW5000. This TF regulates 50 miRNAs in NW1000, which target 66 proteins involved in OC. The vertically arranged miRNAs are all miRNAs that are predicted to be regulated by BRCA1. The horizontally arranged genes are those that are targeted by the miRNA. BRCA1 is itself targeted by *hsa-mir-24-2*.

Figure 2 shows a heatmap illustrating a sub-network of NW1000 concentrating on the TF BRCA1. This node can be regarded as the most significant node for OC relevant miRNA regulation. It is ranked 1^st ^in both networks NW1000 and NW5000. This TF regulates 50 miRNAs in NW1000 (124 in NW5000), which target 66 (115 in NW5000) proteins involved in OC. The left hand side of Figure 2 shows all miRNAs that BRCA1 is predicted to regulate. Across the top all genes are listed that are reported to be targeted by those miRNAs. The hierarchical clustering shown here groups miRNAs and proteins together that display a similar behaviour. The Cyclin-dependent kinase inhibitor 1 (CDN1A) for example is targeted by 13 different miRNAs.

### MicroRNA regulatory network motifs

To better illustrate the cooperation of various nodes in the network we endeavoured to identify network motifs shared by both NW1000 and NW5000 networks. We thus searched for loop-like structures that potentially can be self-promoting, thus contributing to changed expression levels of genes and miRNAs during OC progression. Initially we found five miRNAs in NW1000 (22 in NW5000) that target one of their own predicted TFs.

**Figure 3:**
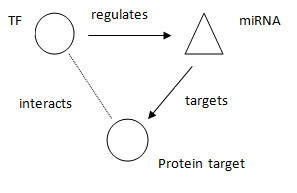
**Loop structure regulatory network motif**. Three-element regulatory loop-structure motif, which features one miRNA and two proteins. In this structure, one of the proteins would act as a TF by regulating the miRNA, which in turn would target the other protein. The latter would either simply interact with the TF or serve as a TcoF for the regulation of the miRNA. This protein would also be associated with OC according to DDOC.

Next we identified three-element network motifs, which feature one miRNA and two proteins. In this structure, one of the proteins acts as a TF by regulating the miRNA, which in turn targets the other protein. The latter either simply interacts with the TF or serves as a TcoF for the regulation of the miRNA. This protein will also be associated with OC according to DDOC [39]. Depending on the observed expression levels of the miRNA in OC, the levels of the protein target would increase or decrease and depending on the type of interaction between the two proteins and depending on the type of TF (activator or repressor), this type of loop structure would either be self-propagating (positive feedback loop) or self-cancelling (negative feedback loop). Figure 3 summarises the structure under consideration. NW1000 possesses 232 different loops of this type, while NW5000 possesses 752 different loops. The full list of loop structures can be found in the Additional Files 5 and 6. In NW1000 (NW5000), a total of 67 (98) TFs, 80 (122) miRNAs and 46 (60) genes relevant for OC progression are involved in these feedback loops.

One interesting protein is the known proto-oncogene MYC that is found up-regulated in many cancers [59]. It has recently been suggested that miRNAs that are regulated by MYC should be targeted as a possible therapeutic strategy [60]. In our network (NW1000) MYC is targeted by 14 different miRNAs and is predicted to regulate 43 miRNAs. According to our analysis MYC is targeted by three miRNAs, which are also predicted to be regulated by MYC. In addition MYC is part of 33 feedback loops. Out of the 46 OC relevant gene targets that are involved in feedback loop structures, a significant majority (31 genes) are involved in the cell cycle regulation pathway. This pathway is highly relevant for cancer development. While in general the enrichment of genes relevant to OC in the cell cycle pathway is quite high (125 out of 379 genes are involved), the enrichment among genes targeted by miRNAs and part of feedback loops is significantly higher. This means that the expression levels of miRNAs in OC could have a significant impact on the cell cycle. For example, the TP53 protein acts as a cell cycle inhibitor. This protein is targeted by six miRNA and is involved in 17 feedback loops of the type described above. Another protein that is involved in the cell cycle regulation pathway is BRCA1 [61]. This protein is involved in 37 possible feedback loops either as a predicted regulator of miRNAs or as a confirmed miRNA target. For other types of cancer it has been shown that changed miRNA expression levels have the potential to affect relevant pathways and to influence disease development [62]. Our analysis shows that miRNAs and their expression potentially play a major role in OC progression through influencing the cell cycle pathway. Another pathway that is highly relevant for the progression of every cancer is apoptosis. We find five OC relevant genes to be involved in this pathway that are also targeted by miRNAs and part of regulatory feedback loops. This points to another potential mechanism, by which miRNAs and their regulation can possibly affect OC development and progression.

## Conclusions

We have established two networks of miRNA in human OC, one network that investigates core promoter elements and one that investigates a more comprehensive set of *cis*-regulatory elements of miRNA regulation. An examination of these networks with a ranking algorithm and a search for loop-structured network motifs reveals some key players in the regulation of miRNA in human OC. Key miRNAs in the progression of OC appear to be *hsa-mir-20a, hsa-mir-24-2, hsa-mir-34a, hsa-mir-21, hsa-mir-17 *and *hsa-mir-hsa-mir-155 *while key TFs are BRCA1, SP1, ESR1, SMAD3, PO2F1 and TFE2 among others.

## Methods

### miRNAs

We considered all human miRNAs that are listed in miRBase [15] and have a confirmed target in DDOC [39].

### MicroRNA targets

We extracted targets for miRNAs from the databases for experimentally verified miRNA targets TarBase [16] and miRTarBase [17]. We only used targets of miRNAs that appeared in DDOC [39].

### Protein-protein interactions

Human PPI were extracted from the following five databases: IntAct [40], BioGRID [41], HPRD [42], Reactome [43], and MINT [44].

### TFs and TcoFs for miRNAs

Proximal promoter sequences (1000bp upstream) and more comprehensive promoter sequences (5000bp upstream) were downloaded from the UCSC Genome browser [47] for the miRNAs. We used binding site models from Biobase Knowledge Library (BKL) [48] to map mammalian TFBSs to the promoter sequences. We only used those TFBSs that are mapped with a core- and matrix score of greater than or equal to 0.9. TFs that were used to create the binding motifs were associated to the TFBS and thus the link TF→ miRNA was created. TcoFs are proteins that interact with TFs. Either they are TFs themselves that do not regulate a miRNA or they are proteins that are not themselves binding to the DNA but are known to interact with a TF and are annotated as participating in transcriptional regulation. We extracted TcoFs from TcoF-DB [49]. Only those TcoFs were extracted that are characterised as 'high-confidence' in TcoF-DB.

### Network node ranking

Our network model is represented as a weighted directed graph [63]. To the best of our knowledge there is no standard procedure for node ranking in weighted directed graphs. Here we define our own method for node ranking, which we believe is appropriate to estimate each node's potential influence in the biological networks described in this article. Our measure for a node in the network shows certain similarities to the Katz centrality measure [64]. However, Katz centrality is only defined for undirected graphs. In addition, we only consider the influence of node *n *to connected nodes up to the second degree, whereas Katz centrality has no such restriction.

Thus, each node *n *in the network is ranked according to its first-degree edges *e*_n1 _(weighted out-degree, sum of weights for directed edges from the considered node) and second-degree edges *e*_n2 _(weighted out-degree, sum of weights for directed edges from the first degree nodes with respect to *n*). The score *S*_n _for a node *n *is specified as:

$$S_{n}=\sum e_{n1}+w*\sum e_{n2},$$

where *w *is a weighting factor for the second-degree edges *e*_n2 _that should have less influence on the score then first-degree edges *e*_n1_.

There are three different types of edges in the network that are differently weighted:

1. miRNA targets protein → *e*_t_

2. TF regulates miRNA → *e*_r_

3. Protein-protein interaction → *e*_i_

In the network model, we specify that *e*_t _= 1, e_t _> e_r _> e_i _> 0. The weight for e_r _is sampled randomly from a uniform distribution between 0 and 1. The weight e_i _is sampled afterwards from a uniform distribution between e_r _and 0. Thus it is ensured that always e_t _> e_r _> e_i _is true. In addition, we sample the weighting factor *w *as well from a uniform distribution between 0 and 1.

With the three random sampled weights we can calculate *S*_n _for each node *n *and rank each node in the network according to *S*_n_. Finally this procedure is repeated 10, 000 times with different randomly sampled weights. The final rank for a node *n *is the average rank over the 10, 000 rankings. This procedure ensures that no fixed weighting schema is used.

## Authors' contributions

SS, US, and VBB conceptualised the study. SS, US, and ME collected and integrated the data, performed the analysis, and wrote the manuscript. All authors read and approved the final manuscript.

## Supplementary Material

Additional file 1 **Network file for NW1000**. S1_NW1000_network.txt contains the complete network NW1000. Click here for file

Additional file 2 **Network file for NW5000**. S2_NW5000_network.txt contains the complete network NW5000. Click here for file

Additional file 3 **Node ranking for NW1000**. S3_NW1000_node_ranking.txt contains the complete node ranking for NW1000. Click here for file

Additional file 4 **Node ranking for NW5000**. S4_NW5000_node_ranking.txt contains the complete node ranking for NW5000. Click here for file

Additional file 5 **Network motifs for NW1000**. S5_NW1000_network_motifs.txt contains all network motifs found in NW1000. Click here for file

Additional file 6 **Network motifs for NW5000**. S6_NW5000_network_motifs.txt contains all network motifs found in NW5000. Click here for file
